# Antibodies against Apoptotic Cells Present in End-stage Lung Disease Patients Do Not Correlate with Clinical Outcome after Lung Transplantation

**DOI:** 10.3389/fimmu.2017.00322

**Published:** 2017-03-21

**Authors:** Kevin Budding, Eduard A. van de Graaf, Tineke Kardol-Hoefnagel, Erik-Jan D. Oudijk, Johanna M. Kwakkel-van Erp, C. Erik Hack, Henny G. Otten

**Affiliations:** ^1^Laboratory of Translational Immunology, University Medical Center Utrecht, Utrecht, Netherlands; ^2^Department of Respiratory Medicine, University Medical Center Utrecht, Utrecht, Netherlands; ^3^Center of Interstitial Lung Diseases, St. Antonius Hospital, Nieuwegein, Netherlands; ^4^Department of Rheumatology, University Medical Center Utrecht, Utrecht, Netherlands; ^5^Department of Dermatology, University Medical Center Utrecht, Utrecht, Netherlands

**Keywords:** lung transplantation, chronic lung allograft dysfunction, antibodies, apoptotic cells, lung endothelial cells

## Abstract

Antibodies against HLA and non-HLA are associated with transplantation outcome. Recently, pretransplant serum IgG antibody levels against apoptotic cells were found to correlate with kidney allograft loss. We investigated the presence of these antibodies in lung transplantation (LTx) patients and evaluated the correlation of pre-LTx serum levels of IgG antibodies against apoptotic cells with LTx outcome. These cells included donor lung endothelial cells (ECs) obtained from lung perfusion fluid collected during LTx procedure. Cells were isolated, expanded *in vitro*, and analyzed as targets for antiapoptotic cell reactivity. Cultured cells exhibited EC morphology and were CD31+, CD13+, and vWF+. End-stage lung disease patients showed elevated serum IgG levels against apoptotic lung EC (*p* = 0.0018) compared to healthy controls. Interestingly, the levels of circulating antibodies directed against either apoptotic Jurkat cells or apoptotic lung ECs did not correlate, suggesting a target cell specificity. We observed no correlation between chronic or acute rejection and pre-LTx serum levels of antiapoptotic antibodies. Also, these levels did not differ between matched patients developing chronic rejection or not during follow-up or at the time of diagnosis, as they remained as high as prior to transplantation. Thus, circulating levels of antiapoptotic cell antibodies are elevated in end-stage lung disease patients, but our data do not correlate with outcome after LTx.

## Introduction

Chronic lung allograft dysfunction (CLAD) is the main complication after lung transplantation (LTx). In 2014, the nomenclature for chronic rejection after LTx has been revised, and CLAD is divided in two different clinical phenotypes, an obstructive form, or bronchiolitis obliterans syndrome (BOS), and a restrictive from, or restrictive allograft syndrome (1). Currently, BOS is the dominant type of CLAD. According to recent reports, the worldwide 5-year BOS free survival rate is 50%, and mean survival post-LTx is around 10 years (2).

The pathogenesis of BOS following LTx is a multifactorial process. Risk factors for BOS include viral infections, primary graft dysfunction, lymphocytic bronchiolitis, air pollution, genetic factors, and episodes of acute rejection (AR) (3, 4). Also, the development of donor-specific antibodies and *de novo* autoantibodies may contribute to the development of BOS, though their role is not fully understood. Some studies have underlined the importance of alloimmune reactions against the transplant, particularly donor-specific HLA antibodies on transplantation outcome (5, 6), although their exact role is debated (7). In addition to alloimmunity, autoimmunity, especially in the form of non-HLA-specific autoantibodies against collagen type V and k-alpha-tubulin, are thought to contribute to an increased risk of BOS development (8, 9). Thus, humoral immunity against the transplant may be important in BOS pathogenesis and progression. Recently, circulating cell death biomarkers are found to be predictive for survival in human LTx, demonstrating the potential importance of the role of apoptosis in complications after LTx (10).

A major limiting factor in kidney transplantation is pretransplant allosensitization, due to blood transfusions, pregnancies, or previous allografts (11). In line with observations in LTx, also preexisting non-HLA antibodies have been associated with an increased risk of rejection. For example, in kidney transplantation, preexisting anti-angiotensin type 1 receptor antibodies and anti endothelin-1 type A receptor antibodies constitute an independent risk factor for graft loss (12, 13). Also, vimentin, an intra-endothelial cell (EC) protein that can be exposed to the immune system after EC damage and can act as a target for antibody formation (14). Interestingly, Gao et al. have shown that pretransplant antibodies against apoptotic Jurkat T cells predict antibody-mediated rejection and graft failure of kidney transplants (15). The antigens of antiapoptotic antibodies have been partly elucidated. Polyreactive antibodies against apoptotic Jurkat T cells may react with phospholipids, phosphatidylserine, and lysophosphatidylcholine, which during apoptosis become exposed on the cell-membrane upon membrane flip-flop (16, 17).

Antibodies against apoptotic cells have also been detected in systemic autoimmune diseases, such as lupus. Indeed, apoptotic cells are considered as far better substrates for autoantibody binding than viable cells (18). Apoptotic bodies display at their cell surface nuclear materials including DNA, chromatin, and ribonucleoproteins. These autoantigens are then accessible to autoantibodies (19). Lastly, it is widely accepted that DNA becomes accessible very early on apoptotic cells, even before phosphatidylserine (20).

Given the similarities in pathogenic mechanisms induced by graft-reactive antibodies in kidney and LTx, we hypothesized that these antibodies against apoptotic targets preexisting or induced upon transplantation may correlate with outcome following LTx. To test this hypothesis we evaluated the presence of circulating antibodies against apoptotic Jurkat cells (anti-AJC) in a cohort of LTx patients and assessed their correlation to outcome. Since ECs are the primary cells encountered by the recipient’s immune system, we also assessed the role of antibodies directed against apoptotic primary lung ECs (anti-AEC) in this respect. The advantage of using primary apoptotic lung ECs is to detect lung EC-specific antibodies. These cells were obtained from the donor during transplantation procedure. Our results indicate that antibodies against both apoptotic EC and Jurkat cells are present in patient serum prior to transplantation, but that these antibody levels do not correlate with transplantation outcome.

## Patients and Methods

### Patients and Sampling

We included LTx patients who underwent LTx within our center between September 2003 and November 2012, and of whom pretransplantation serum was available. Prior to transplantation, patients were assessed for transplant eligibility *via* classical cross-match testing. Pretransplant HLA antibodies were measured *via* the LABScan 100 flow analyzer (One Lambda, CA, USA) or ELISA (LAT, One Lambda), as described previously (7). All patients were treated with standardized immunosuppressive regime consisting of tacrolimus, basiliximab, prednisolone, and mofetil mycophenolate. Patients depicted as being at risk for CMV or EBV reactivation (defined as a CMV−/EBV− patients receiving a graft from a CMV+/EBV+ donor) were prophylactically treated with valganciclovir up until 6 months after transplantation. Informed consent in accordance with the Declaration of Helsinki was obtained from all the patients, and this study was approved by the medical ethical committee of the University Medical Center Utrecht (METC 06-144). All methods were carried out in accordance with the approved guidelines. Serum samples from 20 healthy controls (HC) who donated blood for research purposes were obtained, processed, and stored at −80°C until further usage.

### Perfusate Analysis, Lung EC Collection, and Cell Culturing

To reduce the risk for thromboembolic complication shortly after LTx (21), the grafted lungs were flushed antegradely *via* the pulmonary artery with perfadex solution. During this procedure, the lungs were ventilated at tidal volume and topically cooled. After explantation, the lungs were flushed for a second time, but now *via* the pulmonary vein, until the fluid became clear and upon inspection was free of blood clots. This flush fluid was collected and centrifuged for 10 min at 1,800 rpm. The cell pellet was dissolved in phosphate-buffered saline (PBS) (Sigma-Aldrich, St. Louis, MO, USA) and subsequently loaded on Ficoll-Paque (GE Healthcare, Milwaukee, WI, USA) for cell separation. The cells were isolated from the interphase, put in aliquots of 1 ml in RPMI-1640 (Gibco, Waltham, MA, USA) supplemented with DMSO (Sigma-Aldrich) and 20% v/v fetal bovine serum (FBS) (Bodinco, Alkmaar, The Netherlands), and stored in liquid nitrogen until further processing.

Both whole perfusate and frozen cell aliquots were inspected for the presence of lung EC *via* FACS analysis using a FACSCanto and FACSDiva software (BD Biosciences, Durham, NC, USA) and FlowJo v.10 (FlowJo, LLC, Ashland, OR, USA). Either whole perfusate cell samples or thawed frozen cell aliquots were stained with a combination of CD45-PO (Thermo Fisher Scientific, Rockford, IL, USA), CD31-FITC (BD Biosciences), CD13-PE (BD Biosciences), CD90-BV421 (BioLegend, San Diego, CA, USA), VEGFR-2-AF647 (BD Pharmingen), or respective isotypes (Biolegend and R&D Systems, Minneapolis, MN, USA). Intracellular staining for Von Willebrand factor (vWF) was conducted using vWF-APC (R&D systems) and Intrastain kit (DAKO, Glostrup, Denmark).

For cell culturing, frozen cell samples were thawed at 37°C, washed with RPMI-1640 20% FBS, centrifuged for 10 min at 1,800 rpm, and the pellet was dissolved in EC-specific growth medium (EBM-2, Lonza, Basel, Switzerland) and additives for vascular EC (EGM-2 SingleQuots, Lonza). Cells were cultured in gelatin (Sigma-Aldrich) pre-coated nunclon T-25 culture flasks (Sigma-Aldrich) in EBM-2 supplemented with primocin (Invivogen, San Diego, CA, USA) until confluency. Confluent flasks were treated with accutase (BD Biosciences), and cells were subcultured (50,000 per flask) in nunclon T-75 culture flasks. Cell morphology was assessed *via* light microscopy (Leica DM IL, Leica Microsystems, Wetzlar, Germany), and cell numbers analyzed *via* Bürker–Türk cell counting chambers (Laboroptik Ltd., Lancing, UK).

Jurkat cells (ATCC, Manassas, VA, USA) were cultured in T-75 culture flasks in RPMI-1640 10% FCS supplemented with 1% penicillin/streptomycin (Thermo Fisher Scientific) and subcultured 1:6 twice a week.

### IgG Purification

IgG was purified from frozen serum samples using Magne Protein G Beads (Promega, Madison, WI, USA) according to manufacturer’s instructions. Briefly, 50 μl beads were incubated with 50 μl pre-centrifuged thawed serum samples for 30–60 min at room temperature while shaking. Supernatant was removed *via* magnetic separation of the beads, which were subsequently washed multiple times. Purified IgG was eluted from the beads, and the purified IgG quality and concentration was obtained using the NanoDrop^®^ ND-1000 system (Thermo Fisher Scientific). Purified IgG fractions were stored at 4°C until further usage.

### Antibodies against Apoptotic Jurkat and Primary Lung ECs

Anti-AJC and anti-AEC antibodies were assessed *via* flow cytometry. Jurkat cells were co-incubated with 1 μl of 1 μM staurosporine (Sigma-Aldrich) O/N at 37°C to induce apoptosis. Cells were centrifuged for 5 min at 1,800 rpm, the supernatant discarded, and the cell pellet was dissolved in 5 ml culture medium. One-hundred microliters of cell suspension were added to a 96-well round bottom plate (Sigma-Aldrich), centrifuged (5 min, 1,500 rpm), and subsequently were incubated with 60 μl purified IgG (diluted 1:2 in PBS) for 30 min at 37°C. Each well was washed twice with 150 μl PBS and centrifuged (5 min, 1,500 rpm). The supernatant was discarded, and the cells were incubated with mouse anti human IgG PE (BioLegend), diluted 1:12.5 in PBS for 30 min at 4°C. Cells were washed once with 150 μl PBS and once with 150 μl annexin V binding buffer (1 l H_2_O + 10 mM Hepes + 140 mM NaCL + 2.5 mM CaCl_2_⋅2H_2_O; pH 7.4). Cells were centrifuged, the supernatant discarded, and100 μl binding buffer supplemented with 4 μl 7AAD (BD Pharmingen, San Diego, CA, USA) and annexin V FITC (ITK Diagnostics, Uithoorn, The Netherlands), diluted 1:2, was added followed by 10 min incubation at room temperature and fluorescence measurement.

In order to induce apoptosis, lung EC were subjected to serum starvation for 48–72 h at 37°C, culturing with staurosporine O/N or exposed to UV light using an UV stratalinker (Stratagene, Santa Clara, CA, USA). Serum starvation yielded the largest amount of apoptotic EC (35–40%). After serum starvation, cells were washed with PBS and detached with 4 ml accutase for 5 min at 37°C. Cell detachment was determined *via* light microscopy. Six milliliters of culture medium was added to the culture flask and centrifuged for 5 min at 1,500 rpm. The supernatant was discarded and the cell pellet dissolved in PBS. One hundred microliters cell suspension/well (V bottom 96-well plate, Sigma-Aldrich) and incubated with 60 μl purified IgG (mean concentration 548 ± 154 μg/ml), diluted 1:2 in PBS for 30 min at 37°C. Subsequent antibody incubations were identical to the previous described Jurkat protocol. All measurements for either Jurkat or primary EC were conducted on the same day. To determine the intra- and inter-assay variation, cells were seeded in duplicate and identical sera measured on multiple days. The inter- and intra-assay variability for this assay were 18 and 4.1%, respectively.

### Statistics

The D’Agostino and Pearson omnibus normality test was used to test the data for Gaussian distribution. Differences in not-normally distributed data were analyzed *via* the Mann–Whitney test, whereas Student’s *t*-tests were used to asses differences in normally distributed data. Correlation analyses on non-normally distributed data were performed with the Spearman’s rank correlation coefficient. Statistical analyses were performed using GraphPad Prism version 6.02 (GraphPad Software Inc., San Diego, CA, USA) and SPSS version 20 (IBM Corp., Armonk, NY, USA). Information on statistical testing and graph display is depicted in the respective figure legend. A *p*-value <0.05 was considered to be statistically significant.

## Results

### Patients

A total of 80 patients who underwent LTx within our center between September 2003 and November 2012, and of whom pretransplantation serum was available, were included in this study. Twenty-nine of these patients were diagnosed with BOS based upon ISHLT criteria (22), and in our total cohort 22 patients died during follow-up, of whom 9 were diagnosed with BOS. Seventeen patients developed one or more episodes of AR, diagnosed, since biopsies were unavailable, as a spontaneous decline of FEV_1_ reversible *via* steroid-pulse treatment. One patient was positive for anti-HLA antibodies directed at DQ4, whereas none of the other patients presented with pretransplant anti-HLA antibodies, as described previously (7). Further clinical and demographic parameters are depicted in Table 1.

**Table 1:** **Patient cohort (*n* = 80)**.

Parameters	Values
Gender, *n* (%)	
Male	39 (48.8%)
Female	41 (51.2%)
Age in year (mean ± SD)	44.4 ± 13.2
Follow-up in months (mean ± SD)	78.7 ± 36.7
End-stage lung disease, *n* (%)	
COPD	37 (46.2%)
CF	27 (33.8%)
ILD	16 (20.0%)
BOS, *n* (%)	29 (36.3%)
Months post-LTx BOS (mean, min–max)	41 (5–124)
Deceased, *n* (%)	22 (27.5%)
Months post-LTx deceased (mean, min–max)	40 (8–98)
AR, *n* (%)	17 (21.3%)

*COPD, chronic obstructive pulmonary disease; CF, cystic fibrosis; ILD, interstitial lung disease; BOS, bronchiolitis obliterans syndrome; AR, acute rejection; LTx, lung transplantation*.

### Lung EC Culturing and Characterization

**Figure 1:**
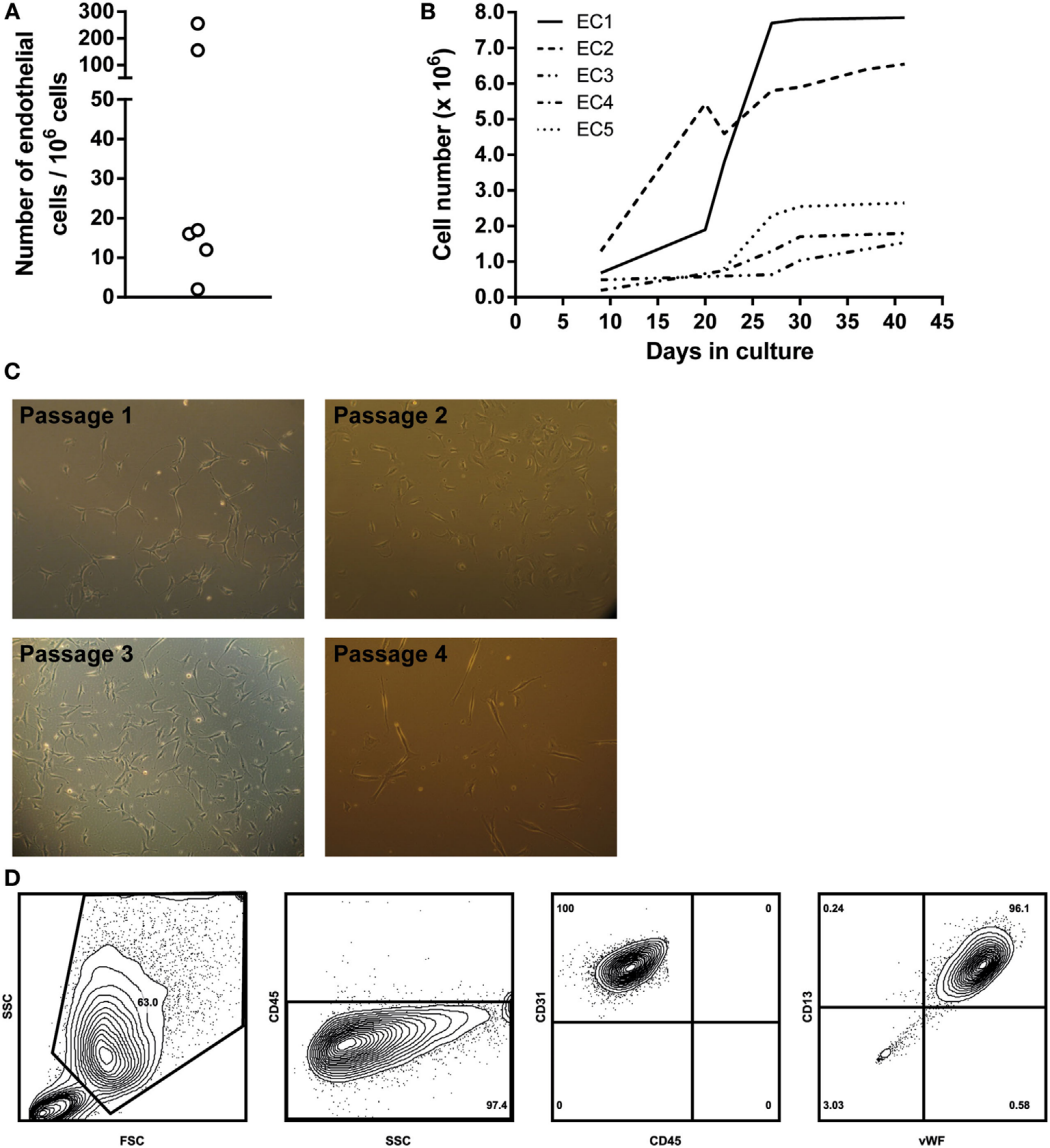
**Endothelial cell (EC) culture characteristics**. **(A)** Schematic representation of the numbers of EC per 1,000,000 cells in a single lung perfusate sample. Cell numbers were quantified *via* flow cytometry. **(B)** Overview of primary lung EC cultures. ECs from different donor perfusate samples were cultured in EBM-2 EC-specific medium. Five different perfusate samples were seeded in gelatin-coated culture flasks, and cell numbers were assessed on different culture days, when cell density was high enough to further passage the cell cultures. Large differences were observed in cell growth rates between individual cell cultures. **(C)** Light microscopy images of different primary EC cultures. Typical EC morphology could be observed, as well as clustering of EC at higher cell densities. Passage 1, 100×, passage 2, 400×, passage 3, 100×, passage 4, 100×. **(D)** EC characterization *via* flow cytometry. Cell cultures were detached using accutase cell detachment solution. ECs were gated as large cells based on forward/sideward scatter. Gated cells were from the non-lymphoid lineage (CD45−) and expressed both CD31 and CD13, characteristic for ECs. Furthermore, intracellular staining revealed these cells to be vWF positive. Density plots were created using FlowJo analysis software.

First, we analyzed perfusate samples from six patients for the presence of vascular EC as determined by FACS. Vascular lung EC were identified as being CD45−/VEGFR-2+/CD13+ and confirmed by intracellular staining of vWF. The percentage of EC in each perfusate was assessed by analyzing 1,000,000 cells. This percentage ranged from 0.0002 to 0.0256% (see Figure 1A). For further analysis of ECs, cells in the perfusate were first expanded in primary cell cultures. Individual ECs were observed within the first week after initial cell seeding. Cell numbers sufficient for subculturing or cell marker analysis were reached in 2–5 weeks (Figure 1B). ECs were cultured for a maximum of nine passages.

Endothelial cells were characterized *via* cell morphology, cell surface, and intracellular markers. Cells in culture presented typical EC morphologic characteristics. Cell density per passage differed. Furthermore, monolayers were observed in later culture passages, and cell morphology remained constant during culturing (Figure 1C, passages 1–4). ECs were non-lymphoid (CD45−). Cells could be cultured without any morphological indications of cell death until passage nine. FACS analysis showed that cultured EC expressed both EC-specific markers CD13 and CD31 on their cell surface. Furthermore, intracellular staining revealed these cells to be positive for vWF (Figure 1D).

### End-stage Lung Disease Patients Exhibit Increased Circulating Levels of Antibodies against Apoptotic Cells

**Figure 2:**
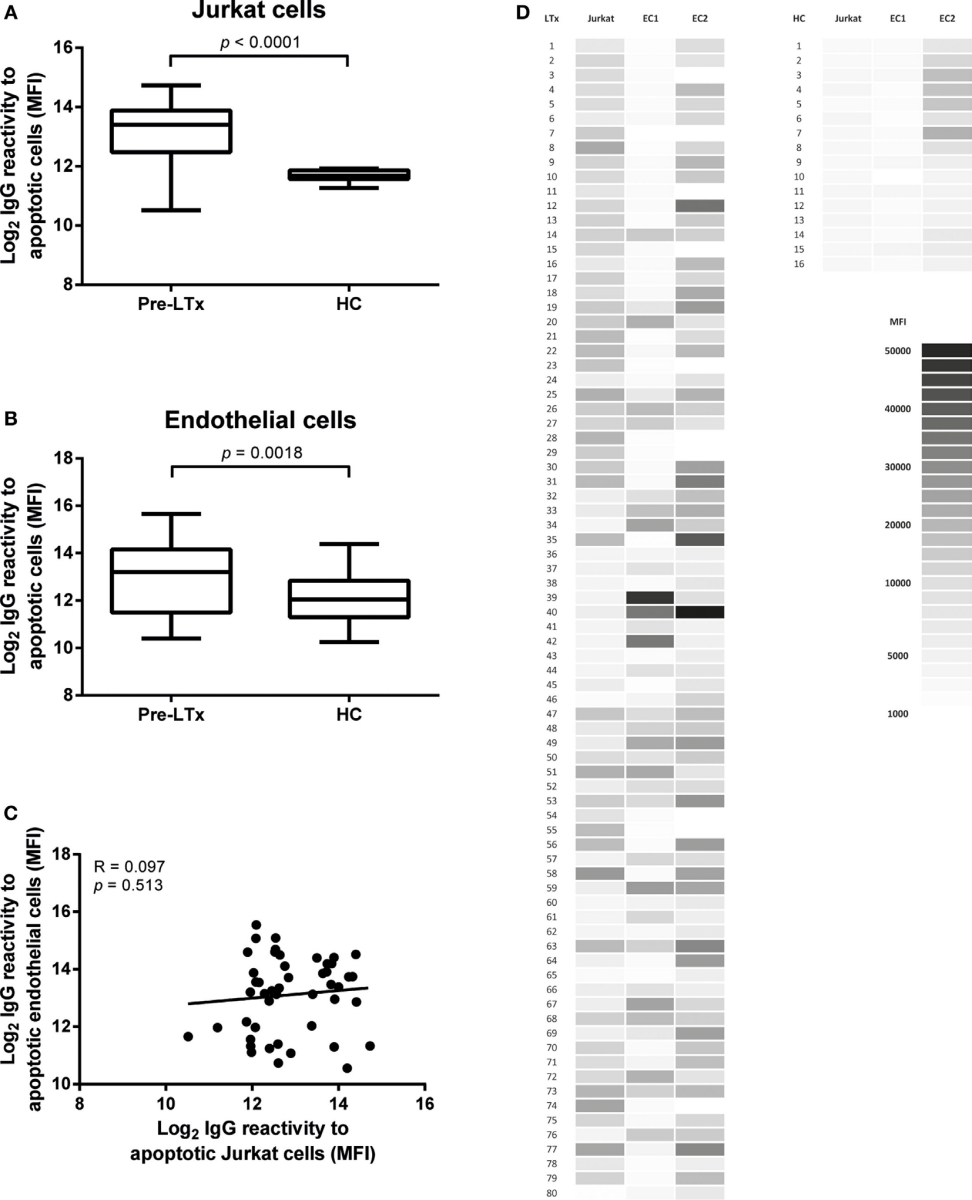
**End-stage lung transplantation (LTx) patients present with elevated levels of serum IgG against apoptotic targets**. Pre-LTx serum IgG reactive against both apoptotic primary endothelial cells (ECs) **(A)** and Jurkat cells **(B)** were measured and compared to healthy controls (HC). End-stage lung disease patients present with significant higher levels of antibodies against apoptotic EC and apoptotic Jurkat cells compared to HC (*p* = 0.0018 and *p* < 0.0001, respectively). The *y*-axis depicts the Log_2_ values of the measured mean fluorescent intensity (MFI). The data are represented as box and whiskers plot where the horizontal bar depicts the median value, the box both 25th and 75th percentiles, and the whiskers the minimum and maximum of the observed values. The data follow a non-Gaussian distribution, Mann–Whitney test, pre-LTx *n* = 80, HC *n* = 20. **(C)** No correlation was found between serum levels of antibodies directed at apoptotic Jurkat or apoptotic ECs. The data are normally distributed, Pearson correlation coefficient. **(D)** Heat-map representation of the 80 measured purified serum IgG samples from LTx patients and 16 HC on both Jurkat cells and 2 lung EC sources. The shades of gray represent the observed MFI according to the scale.

As these cells have been used frequently as a source of apoptotic cells, we first tested the presence of antibodies against apoptotic Jurkat cells. Apoptosis was induced as described in the Section “Materials and Methods.” Overall apoptosis rates in Jurkat cells were around 60–70% of total cell numbers. Higher levels of antibodies against apoptotic Jurkat cells were observed in LTx patients before transplantation as compared to HC (*p* < 0.001, Figure 2A).

Endothelial cells induced to apoptosis were analyzed with flow cytometry. Annexin V+/7AAD− cells were considered to be early apoptotic cells and Annexin V+/7AAD+ cells late apoptotic. Overall apoptosis rates in EC were around 20–30% of total cell numbers. Apoptosis induction *via* exposure to UV radiation did not result in sufficient apoptotic EC numbers (<5%, data not shown). Purified pre-LTx serum IgG of 80 end-stage lung disease patients was analyzed for binding to apoptotic EC, from 2 different lung donors, together with serum IgG of 20 HC. We observed a higher IgG reactivity to apoptotic ECs in pre-LTx serum samples compared to HC (*p* = 0.0018; Figure 2B).

The difference between apoptotic specific IgG levels between end-stage lung disease patients and HCs was higher on apoptotic Jurkat cells. We did not observe differences in IgG antibody reactivity to apoptotic targets when early and late apoptotic ECs or Jurkat cells were differentiated. Stratification of serum IgG titers per end-stage lung disease, clustered as COPD, CF, and ILD did not show any significant differences. Also, no significant differences were observed when age, autoimmune disease, or anti-HLA antibodies were taken into account. Furthermore, we observed no correlation between anti-AJC and anti-AEC (Figure 2C). A heat-map representation of the individually observed mean fluorescent intensity (MFI) values is depicted in Figure 2D.

### Pre-LTx Serum IgG Reactivity to Apoptotic Targets Does Not Correlate with Clinical Outcome after LTx

**Figure 3:**
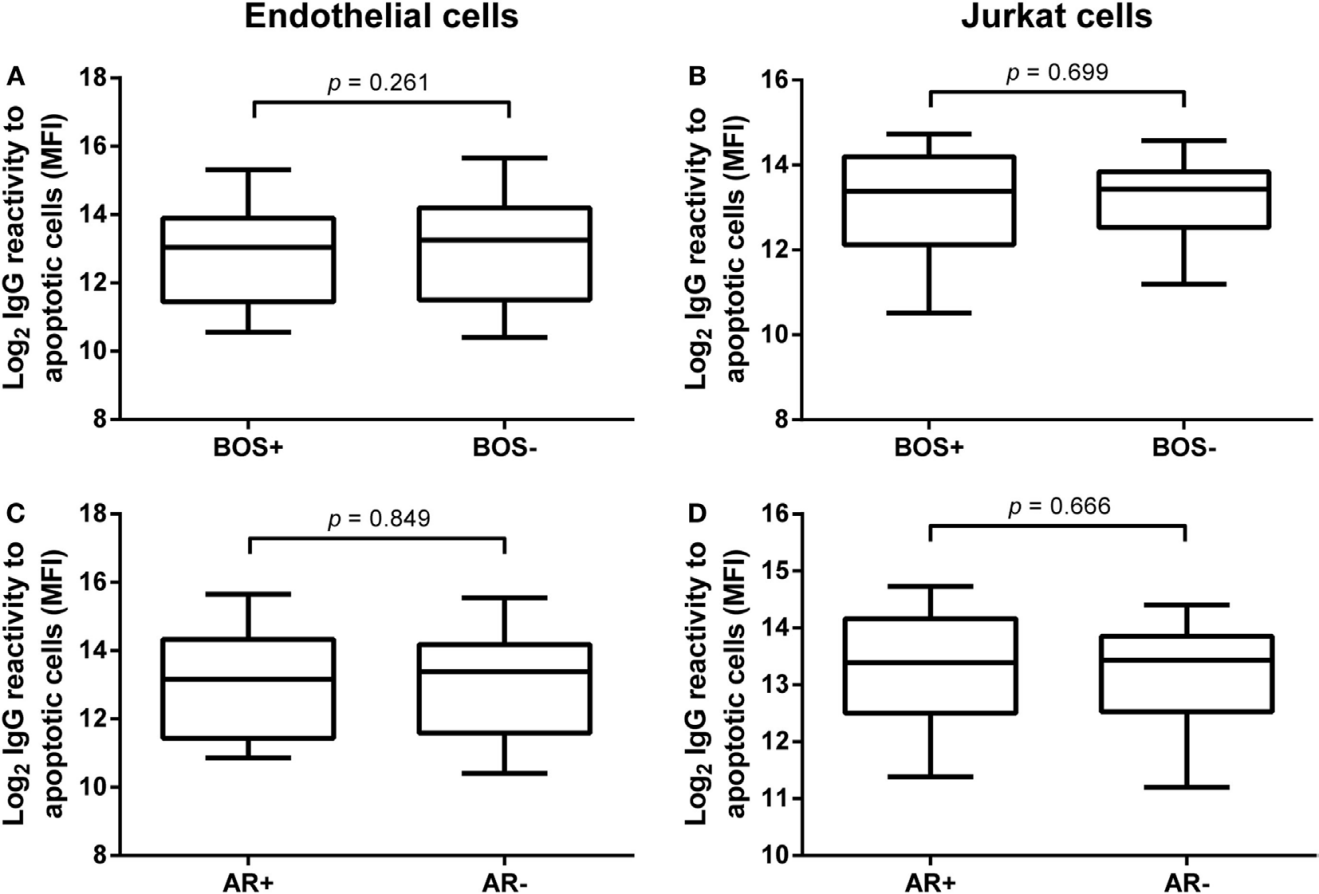
**Pre-lung transplantation (LTx) IgG antibodies to apoptotic cells do not correlate with clinical outcome after LTx**. Pre-LTx purified IgG levels against apoptotic endothelial cells **(A,C)** and Jurkat cells **(B,D)** were stratified according to either bronchiolitis obliterans syndrome (BOS) diagnosis **(A,B)** or the incidence of acute rejection (AR) **(C,D)**. Neither BOS+ nor AR+ patients presented with different purified pre-LTx IgG levels. Therefore, no correlation between IgG reactivity against apoptotic cells and clinical outcome after LTx was observed. The data are represented as box and whiskers plot where the horizontal bar depicts the median value, the box both 25th and 75th percentiles, and the whiskers the minimum and maximum of the observed values. **(B)** Data normally distributed, unpaired *t-*test. **(A,C,D)** Data not-normally distributed, Mann–Whitney test. BOS+ *n* = 29, BOS− *n* = 51, AR+ *n* = 17, AR− *n* = 63.

We analyzed the relation of pre-LTx levels of anti-AJC and anti-AEC and outcome after LTx. First, we stratified patients by development of BOS during follow-up. Twenty-nine patients were diagnosed with BOS. No differences were observed between serum IgG levels of anti-AEC or anti-AJC in BOS+ and BOS− patients (Figures 3A,B, respectively). Also, these pre-LTx levels did not correlate to time to BOS diagnosis or time to death (data not shown).

Second, we investigated the predictive value of pre-LTx serum IgG levels on the incidence of AR post-LTx. In total, 17 patients were diagnosed with one or more episodes of AR. We observed no significant differences between serum levels of anti-AEC or anti-AJC in LTx patients with or without AR (Figures 3C,D, respectively).

### Serum IgG Reactivity during BOS Development

**Figure 4:**
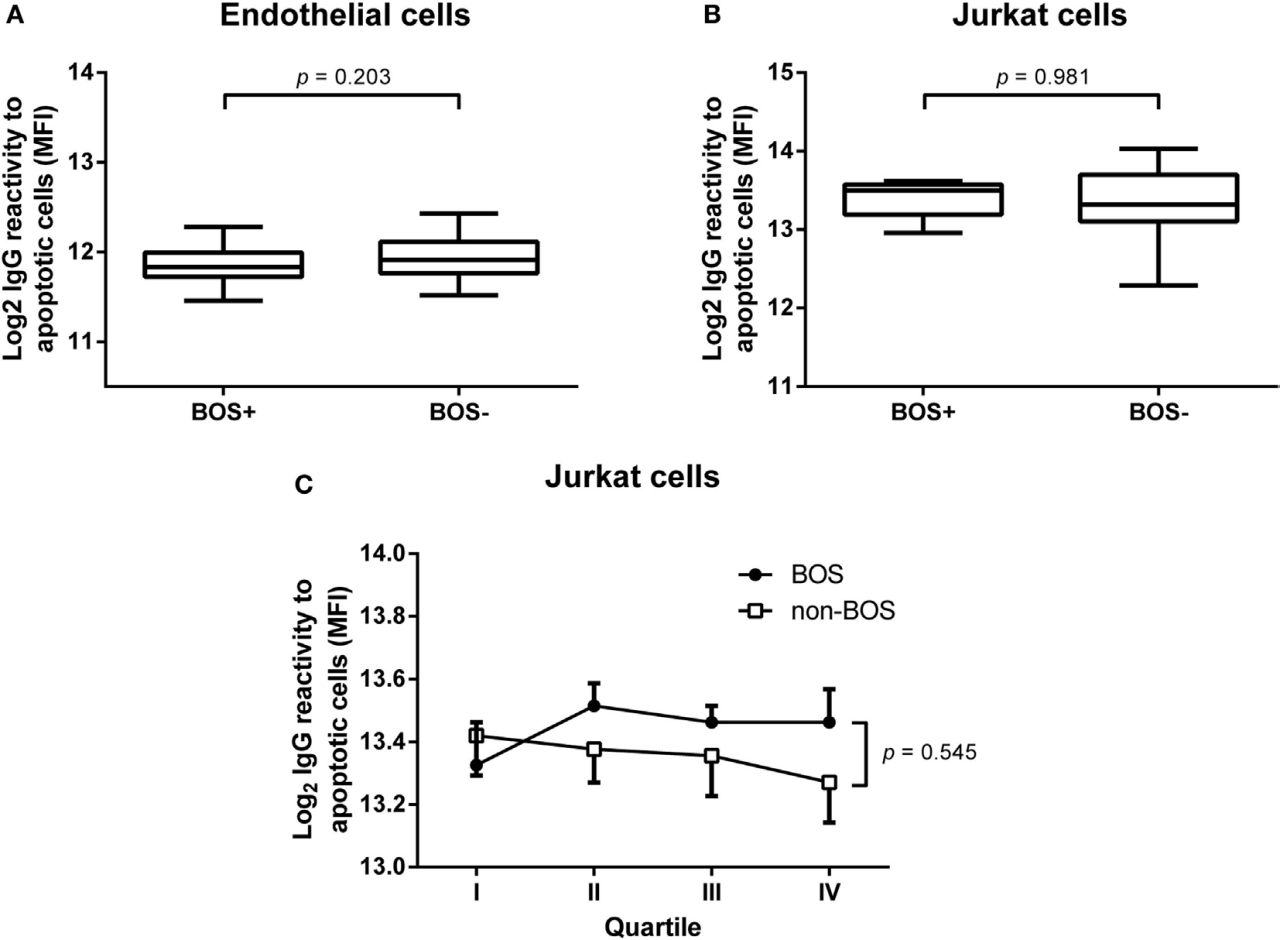
**Purified serum IgG against apoptotic cells at bronchiolitis obliterans syndrome (BOS) diagnosis and progress during transplantation follow-up**. Levels of purified serum IgG, obtained at BOS diagnosis, were analyzed in a cohort of 10 BOS+ patients and 10 BOS− patients matched for age, gender, month after transplantation, and primary lung disease. No differences between these two groups were observed using either apoptotic endothelial cells **(A)** or Jurkat cells **(B)**. Data normally distributed, unpaired *t*-test, BOS+ *n* = 10, BOS− *n* = 10 **(C)**. In order to gain insight into the course of IgG antibodies against apoptotic targets during follow-up after lung transplantation we used a quartile-based selection method. For this end, we divided the time from transplantation to the moment of BOS diagnosis in four equal quartiles and purified IgG from serum taken at each quartile. Parallel to 10 BOS+ patients, we analyzed 10 matched BOS− patients, mentioned above, *via* this approach. BOS+ patients present with slightly elevated IgG levels specific for apoptotic targets, although this difference is not significant. Each dot represents the mean ± SEM of 10 analyzed individuals. Two-way ANOVA on Log_2_ transformed mean fluorescent intensity (MFI), with BOS as independent source of variation.

Since we did not observe any predictive value concerning pretransplant levels of serum IgG reactivity to apoptotic cells, we investigated levels of these antibodies at the time of BOS diagnosis. We isolated serum IgG from serum samples obtained from 10 patients within a month that BOS was diagnosed. In parallel, we selected serum samples from 10 patients who did not develop BOS and were matched for gender, age, primary lung disease, and month post-LTx. IgG was purified from these samples as described in Section “Materials and Methods.” No increase in IgG reactivity was observed against either apoptotic lung EC (Figure 4A) or apoptotic Jurkat cells (Figure 4B).

In order to gain insight into the course of IgG antibodies against apoptotic cells, we divided the time from transplantation to BOS diagnosis in four even time frames (quartile-based selection). Subsequently, we isolated IgG from serum samples obtained during each quartile and analyzed these samples for IgG reactivity. This procedure was also conducted for the matched BOS− patients. Overall, we observed an increased level of IgG antibodies against apoptotic Jurkat cells in BOS+ patient compared to BOS− patients during follow-up. However, this difference is not significant and does not seem to be discriminative at time points prior to clinical manifestations of chronic rejection (Figure 4C).

## Discussion

The prediction and diagnosis of chronic rejection after LTx remains challenging. In this research, we analyzed the levels of antibodies against apoptotic ECs and Jurkat cells in a cohort of end-stage LTx patients. Our results indicate that end-stage lung disease patients have elevated levels of antiapoptotic antibodies against both cell types. However, these titers do not seem to be predictive for the clinical outcome after LTx. Furthermore, we describe the technique to culture primary lung vascular ECs from perfusate obtained during LTx procedure.

Antibodies against apoptotic targets have been identified in previous studies, with Jurkat cells as model for cellular apoptosis (16, 23, 24). In our initial observations on antibodies against apoptotic Jurkat cell we observed Log_2_ IgG reactivity levels in concordance to the results published by Gao et al. Furthermore, the levels of antibodies directed at apoptotic ECs were also within this range.

The primary EC culturing method presented in this paper is novel and has potential implications for LTx research. Donor lung ECs are the first cells recognized by the recipient’s immune cells, initiating, among others, direct allorecognition *via* MHC antigens, which are expressed on these cells (25). Also, ECs are suggested to play a major role in the pathogenesis of chronic rejection, due to the secretion of pro-inflammatory cytokines upon activation (26, 27). Therefore, research has been conducted into the interplay of transplantation patient’s immune cells or serum components, serially collected post-transplantation and EC lines, such as human umbilical vein EC (28, 29). Since our method allows to culture these ECs directly from the donor, this *in vitro* model enables us to investigate the interface of immune activation between donor ECs and patient PBMCs in a biologically more relevant way. This opens new perspectives for LTx research.

Pretransplant antibodies against apoptotic Jurkat cells in kidney transplantation patients have been found to correlate with late kidney allograft loss (15). We did not confirm these observations in our cohort of LTx patients. Neither pre-LTx antiapoptotic IgG serum levels directed against apoptotic ECs nor Jurkat cells correlated with the incidence of acute or chronic rejection. Furthermore, levels of these antibodies during follow-up after LTx were not discriminative for differences in clinical outcome. We also distinguished between early and late apoptotic cells, but no differences could be observed regarding end-stage lung disease or outcome after LTx. These results suggest differences in the contribution of these antibodies on the underlying pathological process in the development of rejection in both kidney and LTx.

Except for one patient, no antibodies against HLA were found in the serum of our cohort, which has been published previously (7). This is in contrast to kidney transplantation cohorts, where highly immunized patients are often observed (15). This low immunization grade can be explained by the fact that no retransplantation patients are present in our cohort. Also, a large part (34%) of our cohort consists of young female CF patients who have not been through pregnancy. Since no anti-HLA antibodies are present, we exclude the possibility that the observed IgG reactivity is directed against either Jurkat or donor allogeneic HLA antigens in both our apoptotic cell death models.

Synergistic effects of anti-HLA antibodies and non-HLA antibodies on graft survival have been described for different non-HLA antibodies, including antibodies against apoptotic cells, in kidney transplantation (30, 31). The impact of anti-HLA antibodies on LTx outcome is less well established as compared to kidney transplantation. Hence, our findings may imply that observations on the impact of various antibodies on graft survival in kidney transplantation cannot be simply extrapolated to LTx. For example, immunosuppressive regimens or tissue-specificity of involved antiapoptotic antibodies, hamper such extrapolation (32, 33).

Clearance of apoptotic cells is facilitated by various immune-involved processes, including IgM natural antibodies, speculated to be specific for lysophospholipids (14, 24). Furthermore, it is postulated that, in the autoimmune disease systemic lupus erythematosus, class-switching of the natural IgM antibodies to the IgG isotype occurs (34). Whereas their epitopes are unknown, antibodies of the IgG isotype against apoptotic Jurkat cells are believed to be polyreactive against multiple epitopes on the membranes of apoptotic cells, including phospholipids (17). The level of apoptosis induction differed between ECs (35%) and Jurkat cells (60–75%), and differences in sensitivity to apoptosis induction has been described previously (35). This difference could affect the absolute number of detected antiapoptotic antibodies. Interestingly, we found no correlation between the levels of antibodies reactive against apoptotic Jurkat cells or apoptotic lung ECs, which could be contributed to a different epitope specificity of these IgG antibodies. The nature of these epitopes, in particular ECs, remains speculative and therefore in-depth antibody characterization and research on subsequent effector mechanisms is expedient.

The importance of antibodies in end-stage lung diseases has been stressed by previous research from our group and others. Autoantibodies have been identified in end-stage CF patients (35, 36), and the levels of various autoantibodies are associated with disease progression (37). Autoantibodies, including antibodies against various tissue epitopes, are present in COPD patients and relate to lung function (38, 39), Indeed, indications for autoimmunity have been observed in specific subgroups of ILD patients (40, 41).

To our knowledge, we are the first to report on antibodies against apoptotic cells in end-stage lung disease patients. We speculate that extensive tissue damage in the diseased lungs of LTx patients has led to the presentation of novel antigens, which have triggered antibody formation. In conclusion, preexisting antibodies against apoptotic lung ECs or apoptotic Jurkat cells do not correlate with outcome after LTx, which is in marked contrast to observations in other transplantation settings. Hence, one should be careful to extrapolate observations on immune mechanisms from one transplantation setting to another.

## Author Contributions

KB and TK-H performed the research; KB, EG, TK-H, CH, and HO participated in data analysis; EG, E-JO, and JE contributed patient material; EG and HO participated in research design; KB, EG, CH, and HO wrote the paper. All authors provided final approval of the version to be published.

## Conflict of Interest Statement

The authors declare that the research was conducted in the absence of any commercial or financial relationships that could be construed as a potential conflict of interest.
