# Supplementary material for: Antibodies against Apoptotic Cells Present in End-stage Lung Disease Patients Do Not Correlate with Clinical Outcome after Lung Transplantation
Source: Front Immunol. 2017 Mar 21;8:322. doi: 10.3389/fimmu.2017.00322 (PMC5359236; doi:10.3389/fimmu.2017.00322)
Supplement: Supplementary file 1 [file Image_1.pdf]

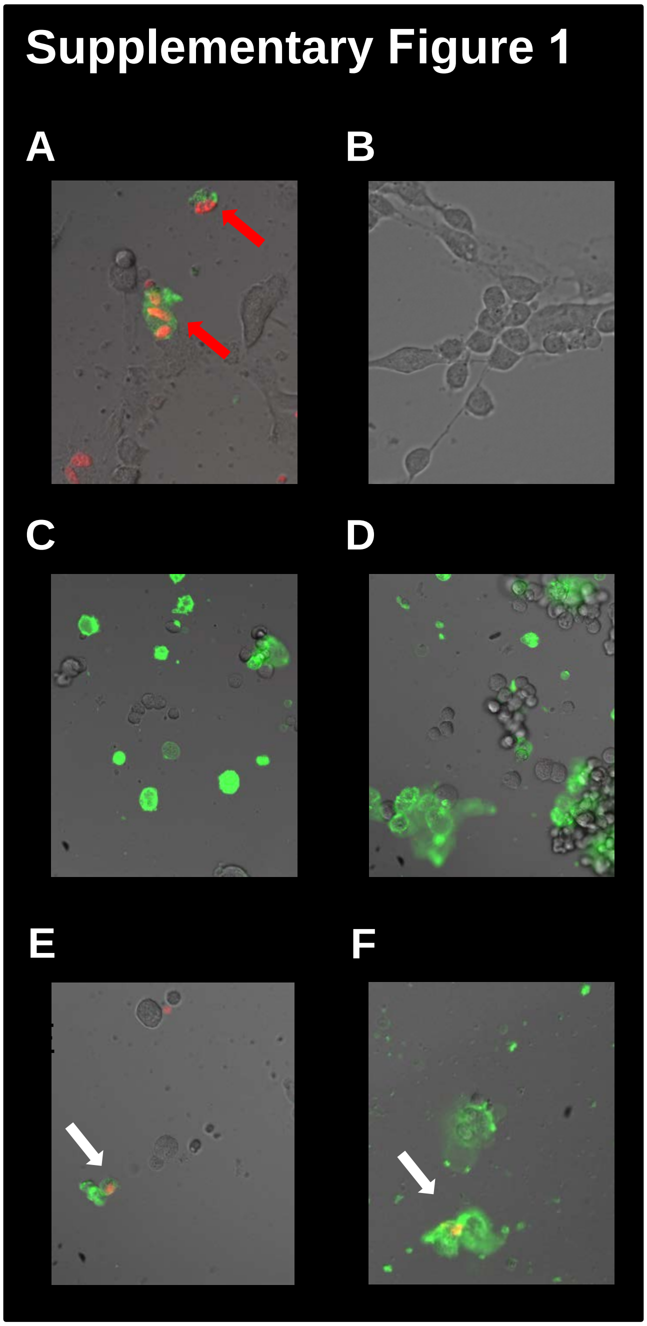

Supplementary Figure 1: Visualisation of anti-apoptotic antibodies via fluorescent microscopy

Lung-derived endothelial cells were cultured in EBM-2 culture medium till 50-75% confluency and subjected to serum starvation (**A**,**C**,**D**,**E**,**F**) or not (**B**) for 72 hr at 37°C. Cells were incubated with Annexin V (green) and 7-AAD (red) to visualize apoptosis (**A** and **B**). Cells were incubated with Annexin V (green), purified human IgG, and mouse anti human IgG PE (red) (**C**,**D**,**E**,**F**). Purified IgGs were isolated from HC serum (**C** and **D**) or LTx patient serum (**E** and **F**). Due to overlap in emission 7-AAD could not be used in combination with mouse anti-human IgG PE. Red arrows indicate Annexin V^+/7-AAD^+ cells and white arrows indicate Annexin V^+/anti human IgG PE^+ cells. Images were acquired using a Leica DMI 4000 B and accompanying software suite (Leica Microsystems, Wetzlar, Germany).
